# Erteng-Sanjie Capsule Enhances Chemosensitivity of 5-Fluorouracil in Tumor-Bearing Nude Mice with Gastric Cancer by Inhibiting Notch1/Hes1 Signaling Pathway

**DOI:** 10.1155/2021/9980565

**Published:** 2021-06-23

**Authors:** Jing Zhou, Shulan Hao, Hao Guo, Han Yu, Zhi Guo, Likun Liu

**Affiliations:** ^1^Department of Oncology, Shanxi Province Hospital of Traditional Chinese Medicine, Shanxi Province Academy of Traditional Chinese Medicine, Shanxi, Taiyuan 030012, China; ^2^Department of Anesthesiology, Shanxi Provincial People's Hospital, Shanxi, Taiyuan 030000, China

## Abstract

Gastric cancer is one of the most common cancers worldwide. This study investigated the chemosensitivity-enhancing effects of Erteng-Sanjie capsule (ETSJC) in combination with 5-fluorouracil (5-FU) on gastric cancer and its possible underlying mechanisms. The study established a subcutaneous xenograft model of human gastric cancer. The animals were divided into five groups: the control group, the 5-FU group, the 5-FU + ETSJC low-dose group, the 5-FU + ETSJC medium-dose group, and the 5-FU + ETSJC high-dose group. The tumor volume and tumor weight were calculated. TUNEL staining was used to evaluate cell apoptosis. Immunohistochemical analysis was used to detect the expression of Ki67^+^ cells and the CD31^+^ microvessel density in tumors. Simultaneously, western blot analysis was applied to detect the expression of caspase-3, Bax, Bcl-2, Notch1, and Hes1 proteins. Compared with the control group, tumor volume and weight in the 5-FU and 5-FU + ETSJC groups were inhibited. Moreover, compared with the 5-FU group, tumor volume and weight were significantly inhibited in the 5-FU + ETSJC groups. The numbers of Ki67^+^ cells, CD31^+^ microvessel density, and the expression of Bcl-2, Notch1, and Hes1 proteins were markedly decreased in the combination group when compared with the chemotherapy alone group. The numbers of TUNEL^+^ cells and the expression of Bax and caspase-3 proteins were significantly increased in the 5-FU + ETSJC groups when compared with the 5-FU group. The therapeutic effects were demonstrated to be dose dependent. In conclusion, the findings of the study showed that ETSJC improved the chemosensitivity of 5-FU by blocking Notch1/Hes1 signaling pathway in gastric cancer-bearing mice.

## 1. Introduction

Gastric cancer has been identified as one of the most common and fatal cancers in the world [1]. More than 70% of the cases (about 677,000) are found in developing countries; of this number, around half are in East Asia [2]. The five-year survival rate for gastric cancer remains very low [3]. For patients with advanced or metastatic gastric cancer, chemotherapy is still the main form of treatment. 5-Fluorouracil (5-FU) is the commonly used drug in chemotherapy [4]; however, chemoresistance and chemotherapy-induced toxicity and side effects make it intolerable for patients [5]. Therefore, therapeutic drugs that can enhance chemotherapeutics but reduce the side effects of chemotherapy are urgently needed.

Traditional Chinese medicine (TCM), which is characterized as “multicomponent-multitarget,” shows its potential in the treatment of gastric cancer. Accumulated evidence has demonstrated that TCM can improve the patients' quality of life as well as prolonging it. It can also prevent the recurrence and metastasis of gastric cancer [6]. Erteng-Sanjie capsule (ETSJC), a patented Chinese medicine, is independently developed by the Shanxi Province Academy of Traditional Chinese Medicine. The main ingredients are: *Pseudostellaria heterophylla*, *Atractylodes macrocephala* Koidz, Coicis Semen, ginger processed *Pinellia ternate*, Citri Reticulatae Pericarpium, *Amomum villosum* Lour, *Gekko japonicus* Dumeril et Bibron, *Sargentodoxa cuneata*, *Smilax china L*, *Vitis quinquangularis* Rehd, Radix *Actinidiae chinensis*, *Prunella vulgaris L*, *concha ostreae*, *Rhizoma curcumae*, *Areca catechu L*, and *Glycyrrhiza uralensis* Fisch. Preliminary clinical studies have shown that ETSJC combined with chemotherapy has significant advantages over chemotherapy alone in alleviating clinical symptoms and reducing the side effects of chemotherapy in patients with advanced gastric cancer [7].

In this study, a subcutaneous xenograft tumor model of gastric cancer was established. ETSJC was administered to investigate its effect in enhancing 5-FU chemosensitivity in gastric cancer.

## 2. Materials and Methods

### 2.1. Cell Culture

A gastric cell line (SGC7901) was purchased from Jennio Biotech Co., Ltd (Guangzhou, China). The cells were cultured in Dulbecco's Modified Eagle's medium (DMEM) containing 10% fetal bovine serum (Gibco, USA) and 1% penicillin/streptomycin, in a humidified 5% CO_2_ incubator at 37°C.

### 2.2. Xenograft Model

Male BALB/c nude mice (4–6 weeks old) were purchased from SPF (Beijing) Biotechnology Co., Ltd. (Beijing, China). 2 × 10^6^ SGC7901 cells were suspended in 100 *µ*L serum-free PBS and were injected subcutaneously into the right flank regions. Tumor volume (TV) was calculated: TV (mm^3^) = *a* × *b*^2^/2 (*a*: length; *b*: width). The experiments were initiated when the tumor volume reached 50–100 mm^3^. The animals were randomly divided into the following: control group, 5-FU group (25 mg/kg/3d), 5-FU (25 mg/kg/3d) + ETSJC low-dose group (0.455 g/kg/d), 5-FU (25 mg/kg/3d) + ETSJC middle-dose group (0.91 g/kg/d), and 5-FU (25 mg/kg/3d) + ETSJC high-dose group (1.82 g/kg/d). The size of the subcutaneous tumors was measured every three days using calipers. At three weeks after treatment, the tumors were removed.

### 2.3. TdT-Mediated dUTP Nick End Labeling (TUNEL) Staining

Paraffin-embedded tumor tissue sections were deparaffinized and rehydrated. To assess cellular apoptosis, the sections were incubated with proteinase K working solution (15 *μ*g/mL in 10 mM Tris/HCl, pH 7.5) for 30 min at 37°C. After washing three times with PBS buffer, they were then incubated with 50 *μ*L of TUNEL reaction mixture, covered with a lid, and incubated for 2 hours at 37°C in the dark. Then, the slides were incubated with 100 *μ*L stopping buffer for 10 minutes and then rinsed in PBS three times. DAPI was applied to detect the nuclei. Images were observed via a fluorescence microscope, and the percentage of the dUTP-positive cells was detected.

### 2.4. Immunohistochemistry

Sections were immersed in 3% hydrogen peroxide for 15 min. Nonspecific antigen blocking was performed in 2% bovine serum albumin (BSA) for 1 hour. The sections were then incubated overnight at 4°C with rabbit anti-CD31 (1 : 200, Bioworld) or rabbit anti-Ki67 (1 : 100, Abcam) and then incubated with biotinylated anti-rabbit IgG (1 : 100, Santa Cruz) for 1 hour at 37°C and with the avidin-biotin-peroxidase complex (1 : 100, Vector Laboratories) for 1 hour at 37°C. Immunoreactivity was visualized with diaminobenzidine staining and imaged under a microscope.

### 2.5. Western Blot

Tissues were homogenized in RIPA plus buffer containing a cocktail of EDTA-free protease inhibitors. The homogenate was centrifuged at 12,000 rpm for 30 min at 4°C. Protein concentration was assayed using the BCA method, then loaded and subjected to electrophoresis in 10% SDS-PAGE gels, and transferred onto PVDF membranes. The membranes were then blocked in 5% BSA for 2 hour and then incubated with one of the following primary antibodies: rabbit anti-caspase-3 (1 : 500, Proteintech) or mouse anti-Bax (1 : 100, Proteintech) or rabbit anti-Bcl-2 (1 : 500, Proteintech) or mouse anti-Notch1(1 : 500, Santa Cruz) or mouse anti-Hes1 (1 : 1000, Santa Cruz), with gentle shaking at 4°C overnight. Then, horseradish peroxidase conjugated goat anti-mouse IgG (1 : 50000, Proteintech) or goat anti-rabbit IgG (1 : 50000, Proteintech) secondary antibodies were incubated with the membranes for 2 hours at room temperature. The immunopositive bands were visualized using an enhanced chemiluminescent substrate (Thermo Fisher) and Bio-Rad ChemiDoc XRS digital documentation system. The amount of protein expression is presented relative to the levels of *β*-actin.

### 2.6. Statistical Analysis

The results of the experiments were expressed as the mean ± standard deviation. Comparisons between groups were assessed by one-way ANOVA, and a *p* value < 0.05 was considered statistically significant.

## 3. Results

### 3.1. Impact of ETSJC on Tumor Growth in the Subcutaneous Xenograft

**Figure 1:**
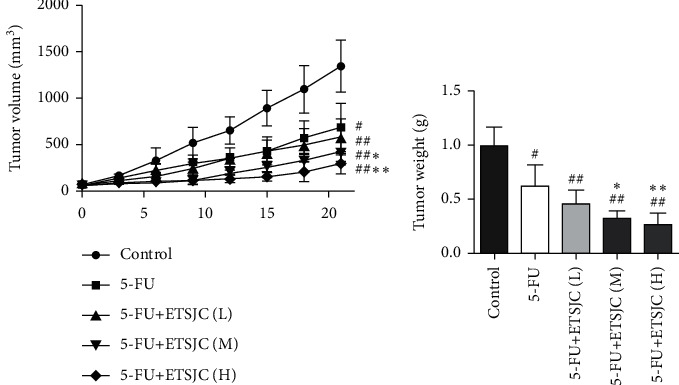
Compared with the control group, the tumor volume (a) and tumor weight (b) of each treatment group were lower. The tumor volume and weight of the 5-FU + ETSJC group were lower than those of the 5-FU group. ^#^*p* < 0.05 and ^##^*p* < 0.01 vs. control group; ^*∗*^*p* < 0.05 and ^*∗∗*^*p* < 0.01 vs. 5-FU group (*n* = 5, per group). ETSJC (L): ETSJC low-dose group; ETSJC (M): ETSJC medium-dose group; and ETSJC (H): ETSJC high-dose group.

To explore whether ETSJC enhanced the chemosensitivity of 5-FU *in vivo*, the study established a subcutaneous xenograft model. As shown in Figure 1, tumor volume (Figure 1(a)) and tumor weight (Figure 1(b)) of the 5-FU and 5-FU + ETSJC groups were significantly reduced when compared with those in the control group. Moreover, the combination of 5-FU and ETSJC produced a significant inhibition of tumor growth compared with animals treated with 5-FU alone. This enhancement effect of ETSJC is dose dependent.

### 3.2. Effect of ETSJC on the Proliferation and Apoptosis in the Experimental Gastric Cancer Model

**Figure 2:**
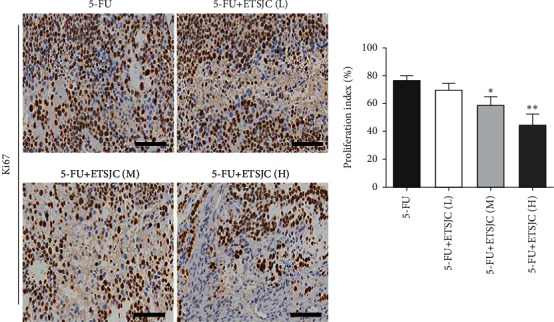
The number of Ki67^+^ cells in the 5-FU + ETSJC group was lower than that in the 5-FU group. ^*∗*^*p* < 0.05 and ^*∗∗*^*p* < 0.01 vs. 5-FU group (*n* = 5, per group). ETSJC (L): ETSJC low-dose group; ETSJC (M): ETSJC medium-dose group; ETSJC (H): ETSJC high-dose group. Scale bar = 100 *μ*m.

The study investigated the effects of ETSJC on tumor cell proliferation and apoptosis. Immunohistochemical analysis showed that a large number of Ki67^+^ cells proliferated in the tumor tissues of the 5-FU group. The number of proliferating cells diminished after the intervention of ETSJC. This diminishing effect of ETSJC is dose dependent (Figure 2).

**Figure 3:**
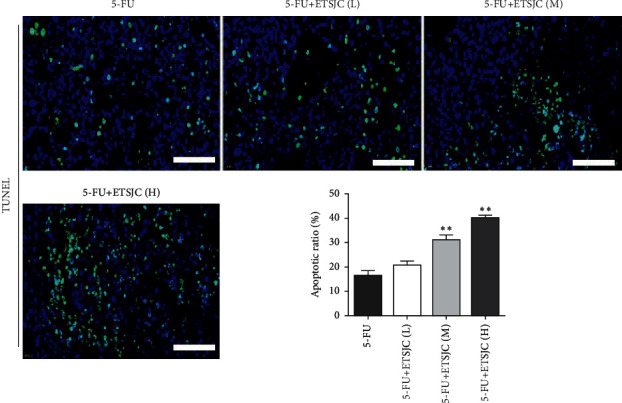
The number of TUNEL^+^ cells in the 5-FU + ETSJC group was higher than that in the 5-FU-treated group. ^*∗∗*^*p* < 0.01 vs. 5-FU group (*n* = 5, per group). ETSJC (L): ETSJC low-dose group; ETSJC (M): ETSJC medium-dose group; ETSJC (H): ETSJC high-dose group. Scale bar = 100 *μ*m.

**Figure 4:**
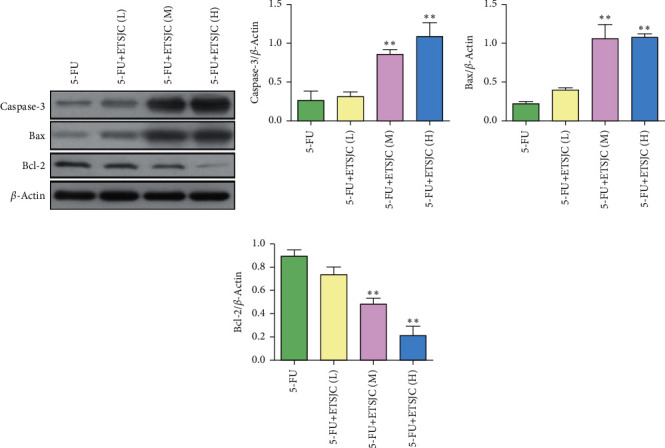
Compared with the 5-FU group, the expression of Bcl-2 protein decreased in the 5-FU + ETSJC group, while the expression of Bax and caspase-3 protein increased. ^*∗∗*^*p* < 0.01 vs. 5-FU group (*n* = 5, per group). ETSJC (L): ETSJC low-dose group, ETSJC (M): ETSJC medium-dose group, and ETSJC (H): ETSJC high-dose group.

Tumor cell apoptosis determined by TUNEL expression assay was used. TUNEL^+^ cells can be found in the tumor tissues of the 5-FU group. Compared with the 5-FU group, the number of TUNEL^+^ apoptotic cells increased in the 5-FU + ETSJC group (Figure 3). Further, the expression of apoptosis-related Bcl-2, Bax, and caspase-3 proteins was also detected. The expression of Bcl-2 protein from the 5-FU + ETSJC group decreased when compared with that of the 5-FU group (Figure 4). However, when compared with the 5-FU group, the levels of Bax and caspase-3 proteins dramatically increased in the combination groups. The apoptotic effect was enhanced when the ETSJC concentration was increased.

### 3.3. Effect of ETSJC on Angiogenesis

**Figure 5:**
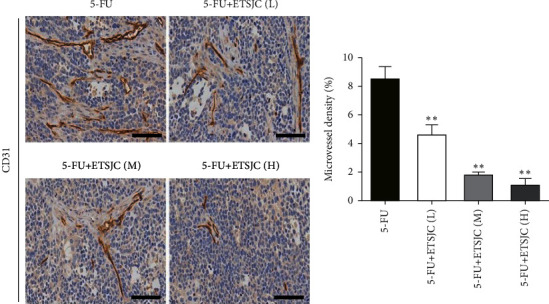
CD31^+^ microvessels in the 5-FU + ETSJC group were fewer than that in the 5-FU group. ^*∗∗*^*p* < 0.01 vs. 5-FU group (*n* = 5, per group). ETSJC (L): ETSJC low-dose group, ETSJC (M): ETSJC medium-dose group, and ETSJC (H): ETSJC high-dose group. Scale bar = 100 *μ*m.

The results showed that the numbers of CD31^+^ microvessels could be observed in the 5-FU group. After combination with ETSJC, the density of CD31^+^ microvessels decreased. The antiangiogenic effect was enhanced with the increase in dose of ETSJC (Figure 5).

### 3.4. Impact of ETSJC on Notch1/Hes1 Signaling Pathway

**Figure 6:**
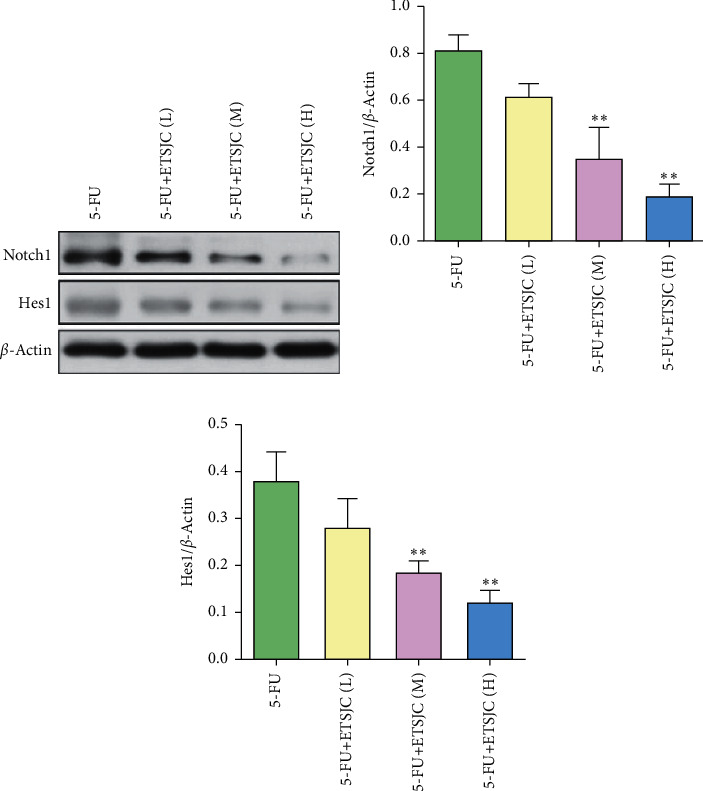
Compared with the 5-FU group, the expression of Notch1 and Hes1 protein in the 5-FU + ETSJC group was reduced. This effect was enhanced by increasing the concentration of ETSJC. ^*∗∗*^*p* < 0.01 vs. 5-FU group (*n* = 5, per group). ETSJC (L): ETSJC low-dose group, ETSJC (M): ETSJC medium-dose group, and ETSJC (H): ETSJC high-dose group.

To further investigate the underlying antitumor mechanisms of ETSJC, the levels of Notch1 and Hes1 proteins were evaluated. Compared with the 5-FU group, the expression of Notch1 and Hes1 proteins in the 5-FU + ETSJC group was reduced. This effect was enhanced with the increasement of ETSJC concentration (Figure 6).

## 4. Discussion

Gastric cancer is one of the most common malignant tumors of the digestive system in the world, and it has attracted more attention in recent years. Chemotherapy remains the main form of treatment for locally advanced and advanced gastric cancer. In chemotherapy, 5-FU is widely used in treating patients with gastric cancer. However, the side effects of chemotherapy often make it difficult for patients to tolerate. For this reason, finding better treatment methods aimed at improving the efficacy of existing anticancer drugs and reducing the toxic side effects is imperative.

Based on the complicated pathological mechanisms of gastric cancer, TCM has shown its efficacy in the treatment of gastric cancer with its “multicomponent-multitarget” characteristics [6, 8]. ETSJC is independently developed by the Shanxi Province Academy of Traditional Chinese Medicine. It is based on Xiangsha-Liujunzi decoction with Chinese medicine. It functions to heat-clearing, detoxifying, stagnation removing, and blood stasis removing. The main ingredients are *Pseudostellaria heterophylla*, *Atractylodes macrocephala* Koidz, Coicis Semen, ginger processed *Pinellia ternate*, Citri Reticulatae Pericarpium, *Amomum villosum* Lour, *Gekko japonicus* Dumeril et Bibron, *Sargentodoxa cuneata*, Smilax China *L*, *Vitis quinquangularis* Rehd, Radix *Actinidiae chinensis*, *Prunella vulgaris L*, *Concha ostreae*, *Rhizoma curcumae*, *Areca catechu L*, and *Glycyrrhiza uralensis* Fisch. ETSJC has the functions of replenishing qi and strengthening the spleen, removing blood stasis and detoxification, and softening the firmness and dispelling lumps. Previous study has demonstrated that ETSJC combined with S-1 plus oxaliplatin (SOX) was better at alleviating the clinical symptoms of patients with advanced gastric cancer than SOX chemotherapy alone. In the current study, a subcutaneous transplanted tumor model of human gastric cancer cells (SGC7901) was established. It was observed that ETSJC combined with 5-FU reduced the volume and weight of the tumor compared with 5-FU alone. The results proved that ETSJC possessed anticancer properties when tested on animals. It enhanced the efficacy of the chemotherapy drug.

Angiogenesis refers to the proliferation and migration of normally static vascular endothelial cells [9, 10]. The aggressive nature of gastric cancer appears to be closely related with the degree of angiogenesis [11]. An increasing number of antiangiogenesis therapy has been extensively developed for cancer treatment, effectively suppressing tumor growth [12, 13]. Apoptosis is a complex multistep process that involves the regulation of the Bcl-2 family, mitochondrial signal transduction, and caspase activation [14, 15]. The caspase protease family is responsible for cell apoptosis. After activation, they further cleave substrates, such as PARP, causing them to lose their normal functions, and increase the activity of the inhibited endonucleases, cleaving the DNA between nucleosomes which eventually lead to apoptosis [16]. The antiapoptotic protein Bcl-2 and the proapoptotic protein Bax are two representative proteins of the Bcl-2 family. Bax and Bcl-2 form a dimer in normal cells. The expression of the two proteins in the cell is relatively stable. When Bax expression increases and Bcl-2 expression decreases, the balance between the two is broken, and apoptosis is induced [17, 18]. The current study found that, after the combined application with ETSJC, the 5-FU + ETSJC group has showed lower CD31^+^ microvessel density than the chemotherapy alone group. The antiangiogenesis effect is obvious. Moreover, the number of TUNEL^+^ cells and the expression of apoptosis-related proteins Bcl-2, Bax, and caspase-3 indicated the apoptosis promoting effects of ETSJC. The combined effects enhanced chemotherapy sensitivity to 5-FU of human gastric cancer cells bearing nude mice. Notch1 signaling pathway is known to regulate the expression of its target genes and plays a key role in cell proliferation, angiogenesis, apoptosis, and differentiation [19–21]. It is well documented that Notch signaling pathway participates in gastric cancer progression [22]. The abnormal activation of Notch signaling is closely associated with tumorigenesis of gastric cancer. Previous study has demonstrated that *γ*-secretase inhibitors (GSIs), which function as Notch signaling inhibitors, combined with 5-FU could be applicable to gastric cancer therapy [23]. In the current study, the expressions of Notch1 and Hes1 were dramatically decreased after combination therapy. This suggested that Notch1/Hes1 signaling pathway was involved in the ETSJC's anticancer feature.

## 5. Conclusions

The study first screened the therapeutic effect of ETSJC on gastric cancer in animal study. The results demonstrated that ETSJC enhanced the chemosensitivity of 5-FU in the subcutaneous xenograft model of human gastric cancer cells. This phenomenon may be related to the inhibition of Notch1/Hes1 signaling pathway; it provides a potential therapeutic method to improve the chemosensitivity of 5-FU in gastric cancer.

## Data Availability

Data are available upon request to the corresponding author.

## References

[1] Siegel R. L., Miller K. D., Jemal A. (2016). Cancer statistics, 2016. *CA: A Cancer Journal for Clinicians*.

[2] Ferlay J., Soerjomataram I., Dikshit R. (2015). Cancer incidence and mortality worldwide: sources, methods and major patterns in GLOBOCAN 2012. *International Journal of Cancer*.

[3] Mitsui Y., Shiina H., Kato T. (2017). Versican promotes tumor progression, metastasis and predicts poor prognosis in renal carcinoma. *Molecular Cancer Research*.

[4] Yamamoto K., Fujiwara Y., Nishida T. (2009). Induction chemotherapy with docetaxel, 5-FU and CDDP (DFP) for advanced gastric cancer. *Anticancer Research*.

[5] Guo X.-F., Liu J.-P., Ma S.-Q., Zhang P., Sun W.-D. (2018). Avicularin reversed multidrug-resistance in human gastric cancer through enhancing Bax and BOK expressions. *Biomedicine and Pharmacotherapy*.

[6] Zhang Z., Li B., Huang J. (2020). A network pharmacology analysis of the active components of the traditional Chinese medicine Zuojinwan in patients with gastric cancer. *Medical Science Monitor*.

[7] Zhang Z. P. (2019). Clinical observation of erteng sanjie capsule combined with sox regimen in the treatment of advanced gastric cancer with spleen deficiency and phlegm stasis syndrome.

[8] Huang Y., Lin J., Yi W. (2020). Research on the potential mechanism of gentiopicroside against gastric cancer based on network pharmacology. *Drug Design, Development and Therapy*.

[9] Folkman J. (1971). Tumor angiogenesis: therapeutic implications. *New England Journal of Medicine*.

[10] Unterleuthner D., Neuhold P., Schwarz K. (2020). Cancer-associated fibroblast-derived WNT2 increases tumor angiogenesis in colon cancer. *Angiogenesis*.

[11] Zhang J.-X., Chen Z.-H., Chen D.-L. (2018). LINC01410-miR-532-NCF2-NF-kB feedback loop promotes gastric cancer angiogenesis and metastasis. *Oncogene*.

[12] Che J., Tao A., Chen S., Li X., Zhao Y., Yuan W. (2016). Biologically responsive carrier-mediated anti-angiogenesis shRNA delivery for tumor treatment. *Scientific Reports*.

[13] Shi H., Jiang J., Ji J. (2014). Anti-angiogenesis participates in antitumor effects of metronomic capecitabine on colon cancer. *Cancer Letters*.

[14] Gu Y.-Y., Chen M.-H., May B. H. (2018). Matrine induces apoptosis in multiple colorectal cancer cell lines in vitro and inhibits tumour growth with minimum side effects in vivo via Bcl-2 and caspase-3. *Phytomedicine*.

[15] Jiao C., Chen W., Tan X. (2020). Ganoderma lucidum spore oil induces apoptosis of breast cancer cells in vitro and in vivo by activating caspase-3 and caspase-9. *Journal of Ethnopharmacology*.

[16] Li X., Su B., Liu R., Wu D., He D. (2011). Tetrandrine induces apoptosis and triggers caspase cascade in human bladder cancer cells. *Journal of Surgical Research*.

[17] Lin B., Li D., Zhang L. (2016). Oxymatrine mediates Bax and Bcl-2 expression in human breast cancer MCF-7 cells. *Die Pharmazie*.

[18] Gobé G., Rubin M., Williams G., Sawczuk I., Buttyan R. (2002). Apoptosis and expression of Bcl-2, Bcl-XL, and Bax in renal cell carcinomas. *Cancer Investigation*.

[19] Zhou W., Wang G., Guo S. (2013). Regulation of angiogenesis via Notch signaling in breast cancer and cancer stem cells. *Biochimica et Biophysica Acta (BBA) - Reviews on Cancer*.

[20] Dang T. P. (2012). Notch, apoptosis and cancer. *Advances in Experimental Medicine and Biology*.

[21] Hibdon E. S., Razumilava N., Keeley T. M. (2019). Notch and mTOR signaling pathways promote human gastric cancer cell proliferation. *Neoplasia*.

[22] Hu J., Yu J., Gan J. (2020). Notch1/2/3/4 are prognostic biomarker and correlated with immune infiltrates in gastric cancer. *Aging*.

[23] Lee H.-W., Kim S.-J., Choi I. J., Song J., Chun K.-H. (2015). Targeting Notch signaling by *γ*-secretase inhibitor I enhances the cytotoxic effect of 5-FU in gastric cancer. *Clinical and Experimental Metastasis*.

